# Taxonomy and Phylogeny of Rust Fungi on Hamamelidaceae

**DOI:** 10.3389/fmicb.2021.648890

**Published:** 2021-04-28

**Authors:** Yun Liu, Bin Cao, Chengming Tian, Yoshitaka Ono, Weiwei Lin, Yingmei Liang

**Affiliations:** ^1^The Key Laboratory for Silviculture and Conservation of Ministry of Education, Beijing Forestry University, Beijing, China; ^2^State Key Laboratory of Mycology, Institute of Microbiology, Chinese Academy of Sciences, Beijing, China; ^3^College of Education, Ibaraki University, Ibaraki, Japan; ^4^Museum of Beijing Forestry University, Beijing Forestry University, Beijing, China

**Keywords:** Pucciniales, *Puccinia*, life cycle, new species and genus, evolution, Asia

## Abstract

Hamamelidaceae is composed of woody plant taxa of important economic value; however, reports on diseases affecting these plants are rare. Three kinds of rusts were studied, of which the first one is characterized by catenulate spores in peridiate columnar sori on *Sycopsis sinensis*, the second one produces two-celled pedicellate teliospores in pulvinate sori on *S. sinensis* and *Corylopsis* spp., and the last one produces aeciospores in cup-shaped aecia on *Hamamelis* spp. Phylogenetic analyses indicated that the three species belong to the same genus in Pucciniaceae. The first fungus is distinct in teliospore morphology, producing one-celled catenulate spores in peridiate columnar sori and molecular phylogeny from species of other genera. Thus, it is described herein as a new genus and species *Novopuccinia sycopsis-sinensis* in Pucciniaceae. The latter two species were reported as *Puccinia corylopsidis* and *Aecidium hamamelidis*, respectively. However, phylogenetic analysis using *ITS* and *28S* genes has revealed that these are closely related to the new genus and species. By combining host, distribution, and evolutionary hypothesis of rust fungi with endocyclic life cycle, these are reclassified as *N. corylopsidis* and *N. hamamelidis*. Taxonomic descriptions, illustrations, and a key to rust fungal species occurring in Hamamelidaceae in Asia are provided.

## Introduction

Several Hamamelidaceae plant species are of significant ecological value in the subtropical humid evergreen broad-leaved forest community (Xu, 2001). Some species such as *Sycopsis sinensis* also have special habitat requirements and therefore show scattered distribution in the whole range of the subtropical humid evergreen broad-leaved forests. *S. sinensis* has been listed as a class of protected species in several provinces (Zang and Bian, 2003; Xu et al., 2007).

Four rust fungi are known to infect trees of Hamamelidaceae in Asia. *Aecidium hamamelidis* Dietel (synonym: *Puccinia mitriformis* S. Ito on *Sasa*) parasitizes *Hamamelis* (Hiratsuka and Sato, 1970; Hiratsuka et al., 1992; Ono, 2017). *P. sasicola* (Hara) Hino & Katum is heteroecious but demicyclic, host-alternating between *Corylopsis* (spermogonial/aecial stages) and *Sasamorpha* (uredinial/telial stages) (Hara, 1939, 1952). *P. corylopsidis* Cummins with two different types of teliospores is microcyclic on *Corylopsis*, and *P. sakamotoi* Hirats. f. & Yoshino with columnar telia is demicyclic and autoecious on *Distylium* (Hiratsuka, 1942).

Rust fungi, Pucciniales, are obligate plant parasites and comprise one of the largest groups in Basidiomycota. Approximately one half of the nearly 8,000 described rust species belong to the genus *Puccinia* Pers., which is mainly characterized by two-celled teliospores (Index Fungorum)^1^. Several molecular phylogenetic studies have shown that *Puccinia* spp. is a polyphyletic group and some species are misclassified under this genus name (Aime, 2006; Minnis et al., 2012; Beenken and Wood, 2015). These recent studies have revealed that the taxonomy of the species within the huge genus *Puccinia* is worth exploring.

In this study, three distinct symptoms with different sporulation of rust fungi were observed on Hamamelidaceae trees. Of these, two fungi produce telia; i.e., one produces two-celled pedicellate spores in pulvinate sori on *S*. *sinensis* and *Corylopsis* sp., while another generates one-celled catenulate spores in peridiate columnar sori on *S*. *sinensis*. Long intercalary cells are prominent in this fungus. The other produces spermogonia and cup-shaped aecia on *Hamamelis* spp. This study aimed to determine the taxonomic status and relationship of three Hamamelidaceae fungi by morphological, molecular phylogenetic, host, and evolutionary analyses of these endocyclic rust fungi. A new genus, *Novopuccinia*, and three species, namely, *N. sycopsis-sinensis* sp. nov., *N. corylopsidis* comb. nov., and *N. hamamelidis* comb. nov., are described and presented here in detail. All of the rust species of Hamamelidaceae are listed, and their hosts and distribution are provided.

## Materials and Methods

### Study Site and Specimens

**FIGURE 1:**
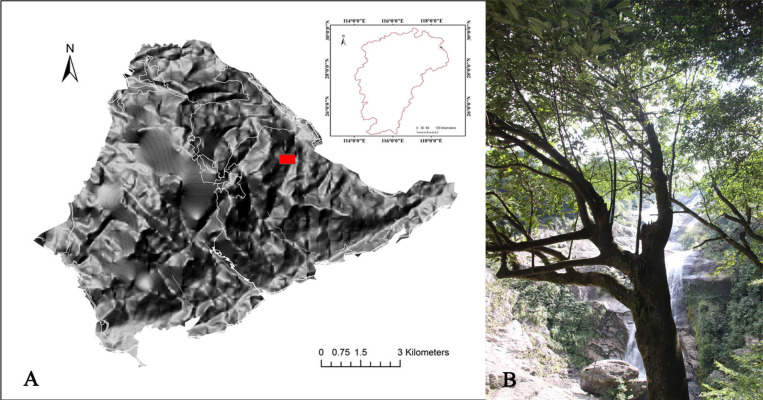
**(A)** Type species location of *Novopuccinia* on Mt. Sanqingshan of Jiangxi Province, China. **(B)** The indigenous host, *Sycopsis sinensis*, occurring to the mountain.

Four rust specimens were collected from diseased *S. sinensis* on Mt. Sanqingshan, northeast Jiangxi Province, China (Figure 1), in 2017. Rust specimens on *Hamamelis* sp. were collected from Ibaraki, Japan, by Y. Ono (IBAR). The host tree was identified by referring to the Flora of China^2^. These specimens were deposited at the Museum of the Beijing Forestry University (BJFC), Beijing, China. Individual specimens are listed under each species in the Taxonomy section. Photomicrographs of type specimens of *P*. *corylopsidis* were provided by the U. S. National Fungus Collections (BPI).

### Morphology

Rust sori from fresh collections were free-hand sectioned using a double-edged blade under a binocular microscope. Spores were scraped from sori on the dried specimens. Thin sections and spores were mounted in a drop of lactophenol solution on a microscope slide and observed, measured, and photographed using a Leica DM3000 upright microscope (Leica, Germany). For scanning electron microscopy (SEM), fragments of dried leaves with sori and spores were mounted on SEM stubs with double-sided tape. The samples were coated with gold using a Hitachi SCD-005 sputter coater and examined with a Hitachi S-3400 scanning electron microscope (Hitachi, Tokyo, Japan) operated at 5 kV.

### DNA Extraction and PCR Amplification

Genomic DNA was extracted from the sori by a method described by Tian et al. (2004). The primer pairs NL1 and NL4 for nuc rDNA 28S (*28S*) (O’Donnell, 1993) and Rust2inv and ITS4rust for Nuc rDNA internal transcribed spacer region (*5.8S-ITS2*) (Aime, 2006; Beenken et al., 2012) were used in this study. The PCR conditions were fully described by Liu et al. (2018). The PCR amplification products were estimated visually by electrophoresis in 1.5% agarose gels. DNA sequencing was performed using an ABI PRISM^®^ 3730xl DNA Analyzer with BigDye^®^ Terminater Kit v. 3.1 (Invitrogen) at the Shanghai Invitrogen Biological Technology Company Limited (Beijing, China).

### Phylogenetic Analyses

The sequences of the *28S* and *5.8S-ITS2* genes from this study and reference sequences obtained from GenBank (Supplementary Table 1) were aligned and edited manually using online MAFFT^3^ (Katoh and Standley, 2013). The alignments were concatenated for phylogenetic analyses. The alignments were concatenated for phylogenetic analyses. Maximum parsimony (MP) analyses were conducted with PAUP v. 4.0b10 (Swofford, 2003), using 1,000 heuristic search replicates with random additions of sequences with the tree bisection and reconnection (TBR) branch swapping algorithm. All molecular characters were unordered and given equal weight; analyses were performed with gaps treated as missing data. All equally parsimonious trees found were saved in the MP analyses. Other calculated parsimony scores were tree length (TL), consistency index (CI), retention index (RI), and rescaled consistency (RC). MP bootstrap analyses with 1,000 replicates were performed in the same way, with 10 rounds of replicates of heuristic search with random addition of sequences and subsequent TBR branch swapping during each bootstrap replicate. ML analysis was performed with a GTR + I + G substitution model selected by MrModeltest v.2.3 (Posada and Crandall, 1998). Branch support was evaluated with a bootstrapping (BS) method with 1,000 replicates. For Bayesian Inference (BI), the best-fit model of substitution among those implementable in MrBayes was estimated separately for each gene using jModeltest (Darriba et al., 2012) on the CIPRES portal, based on the Bayesian Information Criterion (BIC). According to the results of MrModeltest, Bayesian Inference (BI) of the 28S was performed using MrBayes 3.1.2 (Ronquist and Huelsenbeck, 2003), implementing the GTR + I + G model. BI was performed by a Markov chain Monte Carlo (MCMC) algorithm (Rannala and Yang, 1996). The MCMC chains were run for 1 million generations and saved every 1,000 generations. Sequence alignments were deposited at TreeBASE^4^ under the accession number 27,477.

## Results

**FIGURE 2:**
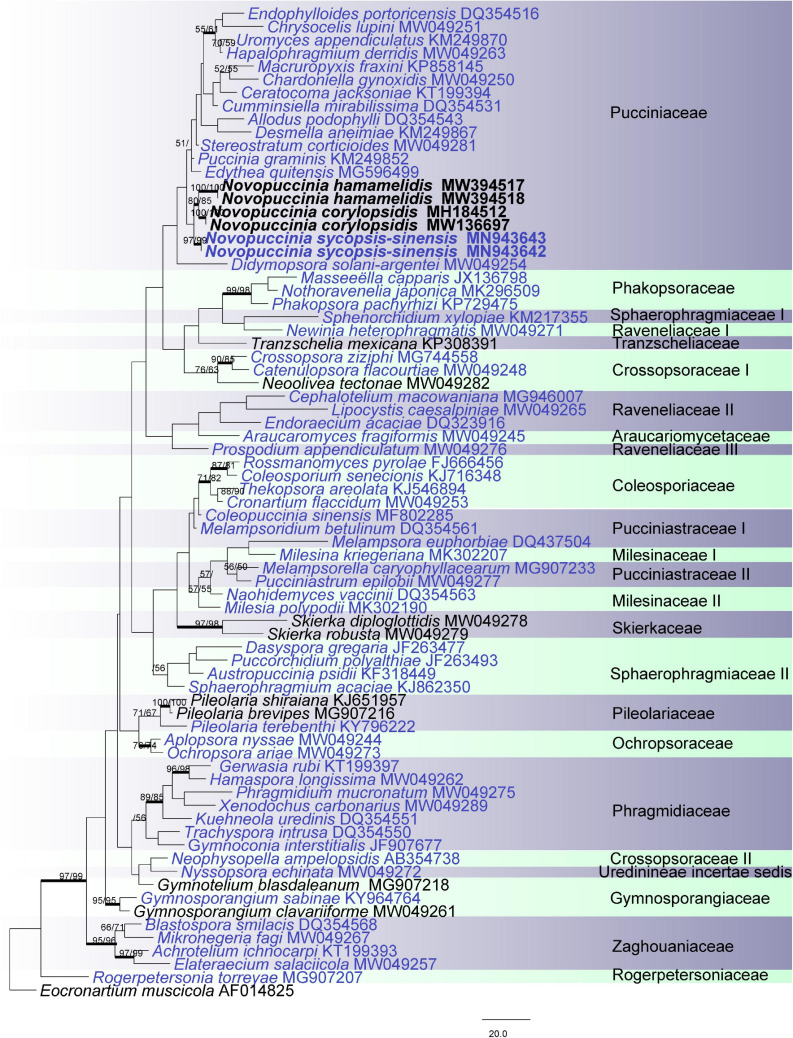
Pucciniales. Phylogram obtained from maximum parsimony, maximum likelihood, and Bayesian analysis of *28S*. The tree is rooted with *Eocronartium muscicola*. Genera represented by types are blue; numbers above branches indicate bootstrapping support (1,000 replicates) for each node as MP/ML. Thickened branches indicate PP > 0.95 from the Bayesian inferences. Bars: 20.0 nucleotide substitutions. The new genus proposed is in boldface.

**FIGURE 3:**
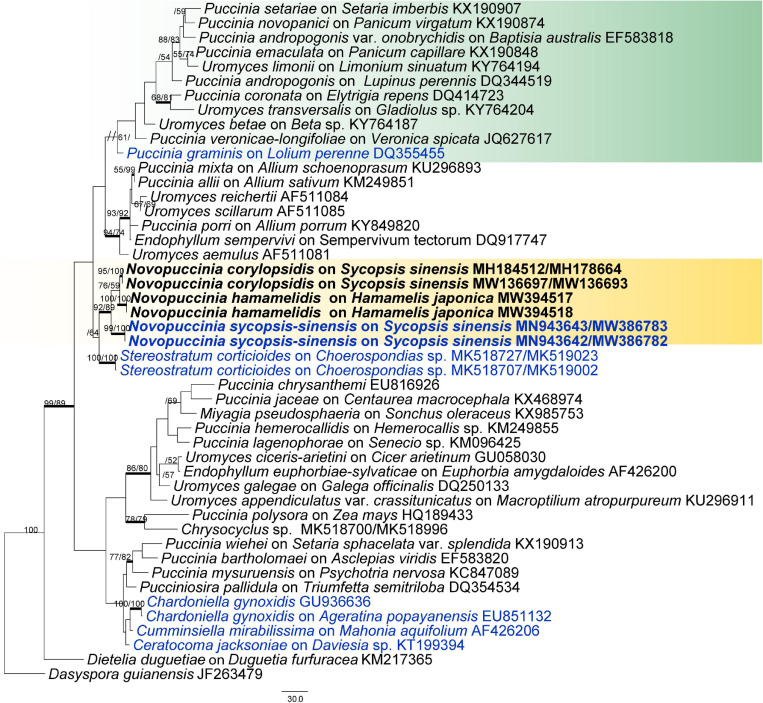
Phylogenetic tree in Pucciniaceae based on maximum parsimony, maximum likelihood, and Bayesian analysis of the *ITS* and *28S* sequences. *Dasyspora guianensis* was used as outgroup. Genera represented by types are blue; numbers above branches indicate bootstrapping support (1,000 replicates) for each node as MP/ML. Thickened branches indicate PP > 0.95 from the Bayesian inferences. Bars: 30.0 nucleotide substitutions. The new genus proposed is in boldface.

To confirm the phylogenetic placement of the fungi in Hamamelidaceae and their relationship, a phylogenetic tree was established based on *28S* (Figure 2). The phylogenetic results were mostly congruent with recent studies of rust fungi (Aime and Mctaggart, 2020). However, the support value was lower because only the *28S* gene was used in the analysis. In this tree, the new genus was clustered with the Pucciniaceae, which was represented by 14 genera and separated from every genus. In addition, the *28S* and *5.8S-ITS2* genes of 46 rust taxa from 13 genera of Pucciniaceae were analyzed, using *Dasyspora guianensis* as outgroup taxa (Figure 3). The MP analyses of the combined *28S* and *ITS* contained 899 characters. Of these, 537 characters were constant, 105 variable characters were parsimony uninformative, and 257 were parsimony informative. There were 11 equally most parsimonious trees, with the first tree (TL = 1,183, CI = 0.496, RI = 0.630, RC = 0.314). The phylogenetic trees obtained from ML and BI analyses with the MCMC algorithm coincided with the MP tree shown in Figure 3. The rust fungi on Hamamelidaceae form three separate clades on one big branch and represent three different species, namely, *Novopuccinia corylopsidis*, *N. hamamelidis*, and *N. sycopsis-sinensis* (Figure 3). The type species of *Puccina*, namely, *P. graminis*, was placed within the *Puccinia*/*Uromyces* subclade. *Novopuccinia* was distantly related to the clade represented by type species *P. graminis* and closely related to *Stereostratum*, with lower support value (Figure 3).

### Taxonomy

***Novopuccinia*** Y. M. Liang & Y. Liu, gen. nov.

MycoBank MB838356

*Type species*: *Novopuccinia sycopsis-sinensis* Y. M. Liang & Y. Liu, sp. nov.

*Etymology*: *Novopuccinia* (Lat.) referring to the new type taxon in *Puccinia*-complex.

*Descriptions*: Spermogonia subepidermal and mostly globose. Aecia subepidermal in origin, cupulate with well-developed peridium (*Aecidium*-type). Uredinia subepidermal in origin and becoming erumpent (*Uredo*-type). Telia subepidermal in origin, becoming erumpent, either pulvinate or columnar; teliospores one-celled and catenulate with a long intercalary cell or two-celled and pedicellate.

*Habitat and distribution*: Currently only known on Hamamelidaceae in Asia.

**FIGURE 4:**
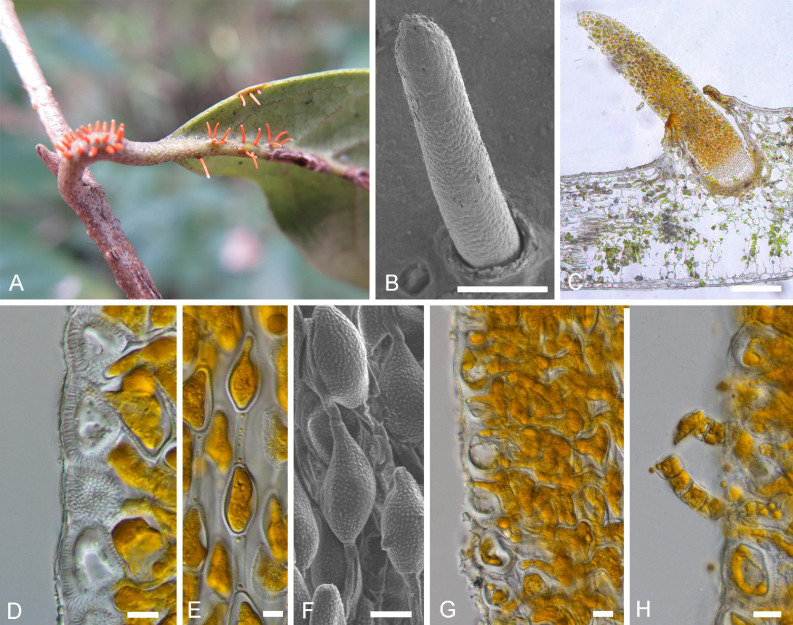
Morphology of *Novopuccinia sycopsis-sinensis* (BJFC-R02976, holotype). **(A)** Sori on a leaf and branches. **(B)** A teliospore, as seen by SEM. **(C)** Longitudinal section of a teliospore, as seen by LM. **(D)** Peridium cells, as seen by LM. **(E)** Teliospores, as seen by LM. **(F)** Teliospores, as seen by SEM. **(G**,**H)** Basidia as seen by LM. Scale bars: **(B,C)** = 1 mm; **(D,H)** = 10 μm.

*Notes*: Morphologically, the telia/teliospores of *Novopuccinia* consist of two types. One is represented by *N. sycopsis-sinensis*, which produces one-celled, catenulate teliospores in columnar telia. It is taxonomically distinct from other morphologically similar genera in Pucciniaceae, i.e., *Ceratocoma*, *Chardoniella*, *Dietelia*, and *Trichopsora*. *Novopuccinia* (Figure 4), *Chardoniella*, and *Trichopsora* produce hair-like telial columns and long, pedicel-like intercalary cells between individual teliospores (Lagerheim, 1891; Kern, 1939). In contrast, *Ceratocoma* and *Dietelia* have small, often inconspicuous, intercalary cells (Buriticá and Hennen, 1980; Buriticá, 1991; Cummins and Hiratsuka, 2003). The presence of a well-developed peridium in the telia is also a distinct characteristic of *Novopuccinia*, separating it from *Ceratocoma*, *Chardoniella*, and *Trichopsora*, which produce non-peridiate telia (Cummins and Hiratsuka, 2003; Berndt, 2018). A metabasidium replaces a probasidium without substantial morphological change in *Trichopsora*, while *Ceratocoma*, *Chardoniella*, and *Novopuccinia* produce a metabasidium external to a probasidium. In addition to these morphological distinctions, *N. sycopsis-sinensis* with one-celled teliospores is clearly separated from *Ceratocoma*, *Chardoniella*, and *Dietelia* in the molecular phylogenetic tree (Figures 2, 3: molecular data of *Trichopsora* for the analysis was lacking).

Another telium/teliospore type is similar to those of *Puccinia* in two-celled with pedicellate (Figure 4), but it can be distinguished from almost all other species by thick-walled teliospores and Hamamelidaceae host. Our phylogenetic results also supported the independence of this type of rust on Hamamelidaceae from the large genus *Puccinia* (Figures 2, 3).

***Novopuccinia sycopsis-sinensis*** Y. Liu & Y. M. Liang, sp. nov. Figure 4

MycoBank MB 838357

*Typification*: CHINA. Mt. Sanqingshan, Jiangxi Province, 28°53′88″N, 118°04′57″E, 1,340 m alt., April 1, 2017, on *Sycopsis sinensis* Oliv, coll. Y.M. Liang (Holotype: BJFC-R02976; Paratype: BJFC-R02983).

*Etymology*: Named after the host name of the type species.

*Descriptions*: Spermogonia, aecia, and uredinia unknown. Telia hypophyllous or petiolicolous, orange, hairlike columnar, exposed, and 2–5 mm long. Teliospores one-celled with elongated pedicel-like intercalary cells (25 μm long), ellipsoid to obovoid, 20–35 × 9–14 μm, and attenuate toward both ends; cytoplasm yellow; the wall colorless, 0.5–1 μm thick, and densely verrucose. Peridium cells rectangular to rounded, 20–31 × 18–23 μm; the wall colorless, 3–5 μm thick, and rough-surfaced.

*Notes*: *N. sycopsis-sinensis* can be easily distinguished from other similar taxa by elongated pedicel-like intercalary cells, one-celled, and peridium cells (Figure 4). According to Tranzschel’s law, the species has an endocyclic life cycle (Buriticá and Hennen, 1980; Buriticá, 1991).

**FIGURE 5:**
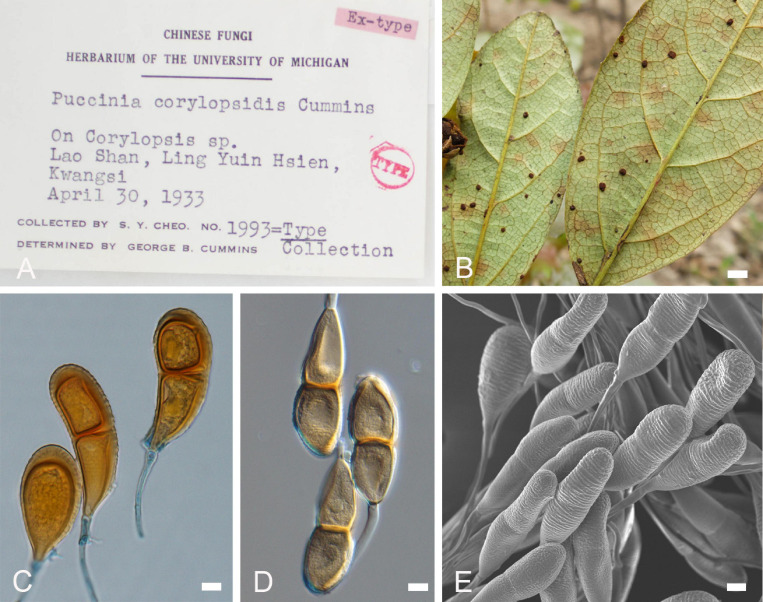
Morphology of *Novopuccinia corylopsidis* (BJFC-R02977, **B–E**). **(A)** Information of type specimen. **(B)** Sori on a leaf. **(C,D)** Teliospores, as seen by LM. **(E)** Teliospores, as seen by SEM. Scale bars: **(A)** = 1 mm; **(B,E)** = 10 μm.

***Novopuccinia corylopsidis*** (Cummins) Y. Liu & Y. M. Liang, comb. nov. Figure 5

MycoBank MB 838358

*Basionym*: *Puccinia corylopsidis* Cummins, Mycologia 43(1): 83 (1951)

*Descriptions*: Spermogonia, aecia, and uredinia unknown. Telia mostly hypophyllous, round, pulvinate, 0.1–0.4 mm in diameter, and blackish brown. Teliospores variable in shape, wall thickness, and surface structure, including two types. One kind was regarded as thin side wall and apex wall slightly thickened or not thickened, 1–2 μm thick, light yellow, oval, 47–65 × 16–20 μm, not or hardly constricted at septum, the wall nearly smooth. The other kind was characterized by the wall apically and unilaterally thickened, 1.5–9 μm thick at sides, up to 18 μm thick at apex, mostly fusoid to oval, 46–82 × 16–29 μm, not or hardly constricted at septum, the wall rugose or densely covered with irregular verrucous wrinkles, and yellowish to brown; the pedicel hyaline, thick-walled, persistent, and up to 150 μm. One-celled spores rarely seen. All these variant spores are produced in the same sorus.

*Habitat and distribution*: On *Sycopsis sinensis* and *Corylopsis* sp. in China (Cummins, 1951; Zhuang et al., 2003).

*Specimens examined*: China. Lao Shan et al., 1931, April 30, on *Corylopsis* sp. (type: BPI 058871); Sanqingshan, Jiangxi Province, 28°53′95″N, 118°04′52″E, 1,342 m alt. (BJFC-R02977) and 28°53′88″N, 118°04′57″, 1,340 m alt. (BJFC-R02978), April 1, 2017, on *Sycopsis sinensis* Oliv., coll. Y.M. Liang.

*Notes*: The species was first reported on *Corylopsis* plants and named *P. corylopsidis* based on *Puccinia*-like teliospores (Cummins, 1951). However, it differs from other *Puccinia* species by dimorphic teliospores (Figure 5). Our molecular phylogenetic analysis indicated that *P. corylopsidis* closely related to *Novopuccinia*, with strong support (Figures 2, 3), hence the new combination in the new genus. *Sycopsis sinensis* is the new host found for *N*. *corylopsidis* in this study.

**FIGURE 6:**
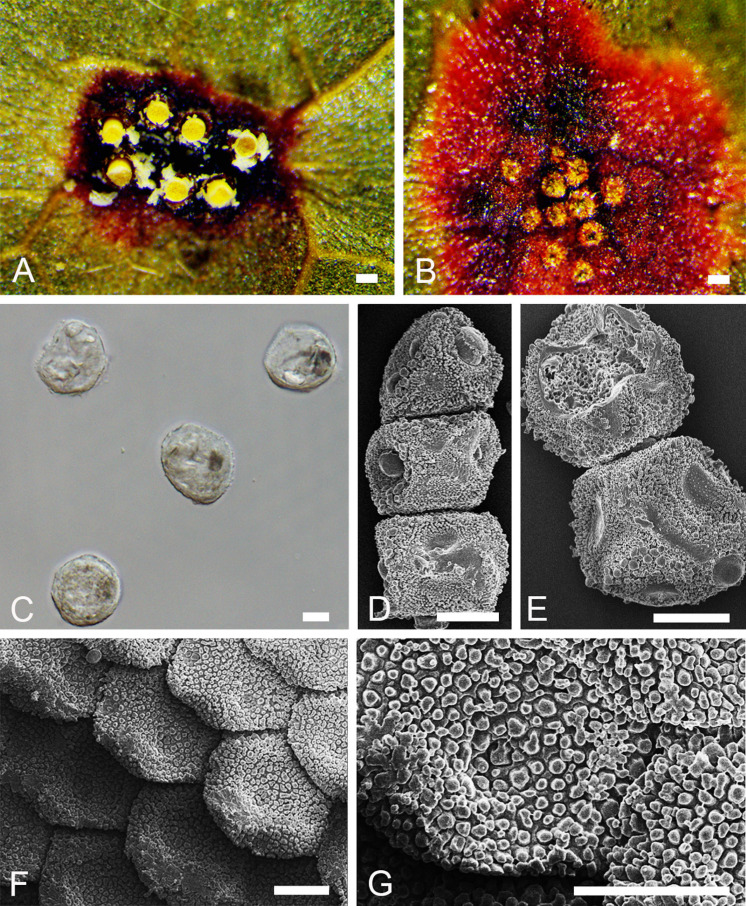
Morphology of *Novopuccinia hamamelidis* BJFC-R3798 (IBAR6666). **(A)** Aecia on a leaf. **(B)** Spermogonia on a leaf. **(C)** Aeciospores, as seen by LM. **(D,E)** Aeciospores, as seen by SEM. **(F,G)** Peridial cells, as seen by SEM. Scale bars: **(A,B)** = 100 mm; **(C,G)** = 10 μm.

***Novopuccinia hamamelidis*** (Dietel) Y. Liu & Y. M. Liang, comb. nov. Figure 6

MycoBank MB 838359

*Basionym*: *Aecidium hamamelidis* Dietel (1899), Bot. Jb. 27: 571

*Synonyms: Puccinia mitriformis* Ito (1909), J. Coll. Agric. Tohaku Imper. Univ. 3(2): 233

*Aecidium hamamelidis* Magnus (1901), Ber. Dt. Bot. Ges. 19: 297

*Descriptions*: Spermogonia and aecia occur on species of *Hamamelis*; spermogonia abaxial, round, 140 μm in diameter. Aecia hypophyllous, cup shape, the cross-section diameter is about 140–150 μm. Aeciospores obovoid or globose, 20–32 × 28–30 μm, surface is covered with irregular verrucosa. Peridial cells round, 30 μm in diameter, outer walls smooth, inner walls moderately rugose with regular papillae. Telia on abaxial surface of *Sasa* plants, early exposed, chocolate-brown; teliospores 95–130 × 18–22 μm, walls usually unilaterally thickened, 2–3 μm on the thin side, somewhat to much thicker on opposite side, 40–70 μm apically, golden brown but the apical thickening much paler, smooth, or perhaps minutely rugose on the broad part of the spore; pedicels mostly not collapsing, colorless, long and tapering, 250 μm long; one-celled spores rare (For telia stage, cited from Hiratsuka et al., 1992).

*Habitat and distribution*: On *Hamamelis japonica* (spermogonial/aecial stages) and *Sasamorpha* spp. (uredinial/telial stages) in Japan.

*Specimens examined*: JAPAN. Ibaraki et al. (1992), August 10, on *Hamamelis japonica* Siebold and Zucc (1992) and Higuchi et al. (1998) (epitype: BJFC-R3798 = IBAR-6666) and June 28, (BJFC-R3807 = IBAR-6615); June 7, on *Hamamelis japonica* Siebold & Zucc., coll. Y. Ono & K. Ishimiya (BJFC-R3799 = BAR-8075); Ibaraki et al. (1994), May 13, on *Hamamelis japonica* Siebold & Zucc., coll. Y. Ono (BJFC-R3800 = IBAR-7170); August 13, 1992, on *Hamamelis japonica* Siebold & Zucc., coll. K. Higuchi, K. Suganuma, and K. Hashimoto (BJFC-R3801 = IBAR-6553); Aomori, Mt. Hakkodasan, July 31, 1992, on *Hamamelis japonica* Siebold & Zucc., Isono (1973) (BJFC-R3802 = IBAR-5567); Gumma, Tone-gun, June 18, on *Hamamelis japonica* Siebold & Zucc., coll. Y. Ono (BJFC-R3803 = IBAR-1585); Tochigi et al. (1992), July 15, on *Hamamelis japonica* Siebold & Zucc., coll. Y. Ono (BJFC-R3804 = IBAR-2194); Ibaraki et al. (1989), July 27, on *Hamamelis japonica* Siebold & Zucc., coll. Y. Ono & H. Miura (BJFC-R3805 = IBAR-3291); Tochigi et al. (1990), August 26, on *Hamamelis japonica* Siebold & Zucc., coll. M. Sawamura and N. Matsumoto (BJFC-R3806 = IBAR-5475).

*Notes*: Dietel (1899) described this fungus as *Aecidium hamamelidis* based on the *Aecidium-*type Aecia on *Hamamelis* plants. It is also related to the bamboo rust *P. mitriformis* S. Ito reported by Hiratsuka and Sato (1970). We did not observe the type specimen of *A. hamamelidis*, but specimens that were collected from the same region and host with type collection were researched. For further study, here we designate BJFC-R3798 as the epitype specimen. Our molecular phylogenetic analysis recovered *A. hamamelidis* within the new genus, with strong support (Figures 2, 3), hence the new combination in *Novopuccinia*.

## Discussion

In this study, three rust taxa obtained from Hamamelidaceae were identified based on morphologic characters and phylogeny based on the *28S* and *5.8S-ITS2* sequences. As a result, *Novopuccinia* typified with *N. sycopsis-sinensis* is proposed as a new genus in Pucciniaceae. The two known species, *P. corylopsidis* and *Aecidium hamamelidis*, were combined to the new genus (*N. corylopsidis* and *N. hamamelidis*) by the same host plant, the molecular data, and endocyclic life cycle.

*Novopuccinia* includes two different teliospore types. The first type is represented by type species *N. sycopsis-sinensis* with one-celled teliospores and belongs to the kind of taxa, whose telia are cupulate, usually with peridia, and teliospores are verrucose and catenulate with intercalary cells. These taxa are considered to have been derived from a *Puccinia*–*Uromyces* complex by reduction of the life cycle through the endophylloid pathway; their occurrence on the same host plants may nonetheless provide clues on the relationship between microcyclic and long-cycled autoecious rust fungi (Jackson, 1931; Buriticá and Hennen, 1980). Members of this complex are closely related to the environment and occur mainly in tropical and other warm regions (Berndt, 2018). The second type is similar to *Puccinia*-like teliospores and represented by *P. corylopsidis* and *Aecidium hamamelidis* (synonym*: Puccinia mitriformis*), but phylogenetically closely related to *Novopuccinia sycopsis-sinensis* (Figures 2, 3). While teliospore characters are deemed important to distinguishing genera and families within Pucciniales (Cummins and Hiratsuka, 2003), molecular phylogenies show that their morphological characteristics may not always define higher taxa (Aime, 2006; Beenken and Wood, 2015). Consequently, several genera have diverged from the genus *Puccinia* sensu lato, despite their similarity in two-celled teliospore morphology such as *Allodus* (Minnis et al., 2012), *Austropuccinia* (Beenken, 2017), and *Puccorchidium* (Beenken and Wood, 2015). However, genera that have been separated from *Puccinia* based on differences in their morphology appear within *Puccinia*, such as *Uromyces*, *Diorchidium*, *Cumminsiella*, and *Miyagia* (Figures 2, 3; Aime, 2006; Van Der Merwe et al., 2008; Beenken and Wood, 2015). New combined species, *Novopuccinia corylopsidis* and *N. hamamelidis*, were defined by combining their distant relationship to type species of *Puccinia* clade, their close relationship with type species *N. sycopsis-sinensis* (Figures 2, 3), common hosts, and evolution hypothesis of the endocyclic life cycle.

The new genus is sister to *Stereostratum* (Figure 3), but they differ in teliospore characters. *Novopuccinia* contains two teliospore types (Figures 4, 5), while *Stereostratum* has one kind of teliospore (Mangus, 1898). Teliospores of *N. corylopsidis* and *N. hamamelidis* are thick-walled and have one germ pore in each cell (Figure 5; Hiratsuka and Sato, 1970; Hiratsuka et al., 1992), while *Stereostratum* spp. have three germ pores in each teliospore cell, and their wall does not thicken (Mangus, 1898).

The type species of *Novopuccinia*, namely, *N. sycopsis-sinensis*, is microcyclic in the life cycle and produces catenulate, rough-surfaced teliospores with intercalary cells between them. The similar life cycle pattern and morphological characteristics and close phylogenetic relationship to *N. corylopsidis* and *N. hamamelidis* (Figures 2, 3) indicate that *N. sycopsis-sinensis* was derived from a hypothetical puccinioid ancestor by reduction of the life cycle through the endophylloid pathway (Jackson, 1931; Buriticá and Hennen, 1980). In this pathway, morphologically *Aecidium*-type telia of a derived microcyclic species are produced on a spermogonial/aecial host plant of a putative ancestral macrocyclic heteroecious species. Similarly, *N. corylopsidis* might have been derived from the same hypothetical ancestor by reduction of the life cycle through the telioid pathway (Jackson, 1931; Buriticá and Hennen, 1980). The latter evolutionary pathway is well-known and is accounted for by Tranzschel’s Law (Shattock and Preece, 2000). These two evolutionary phenomena and resulting correlations between macrocyclic heteroecious species and microcyclic species have been corroborated to a slight extent by a molecular phylogenetic method. Early successful studies include graminicolous puccinias (Zambino and Szabo, 1993), macrocyclic heteroecious *Cronarium* and endocyclic *Peridermium* (Vogler and Bruns, 1998), the *P. monoica* species complex (parasitizing on Brassicaceae and Poaceae) (Roy et al., 1998), and the *P. hemerocallidis* species complex (parasitizing Caprifoliaceae and Xanthorrhoeaceae) (Chatasiri et al., 2006).

*Sycopsis sinensis* (Hamamelidaceae) has been listed as a protected species in China based on its ecological value and rarity (Zang and Bian, 2003; Xu et al., 2007). No disease has been reported in this species to date. Two distinct rust fungi on two adjacent *S. sinensis* trees, *N. sycopsis-sinensis* and *N. corylopsidis*, were found in the present study. *N. corylopsidis* has earlier been reported on *Corylopsis* sp. (Cummins, 1951), and now we observe host expansion and parasitism involving *S. sinensis*. At the same time, we suspect that the species had mutated to adapt to the environment, thus forming a new rust *N. sycopsis-sinensis* that also causes disease.

In addition to these three species, two more species have been reported on Hamamelidaceae in Japan, namely, *P. sasicola* (Hara) Hino & Katumoto on *C. glabrescens* (spermogonial/aecial stages) and *Sasamorpha borealis* (telial stage), *P. sakamotoi* Hirats. f. & Yoshin on *Distylium* spp. (Hiratsuka, 1942; Hino and Katumoto, 1955). The former is heteroecious and demicyclic; its teliospores are thick-walled, similar to those of *N. corylopsidis and N. hamamelidis* (Hino and Katumoto, 1955; Hiratsuka et al., 1992). However, it differs from the latter two species by its demicyclic life cycle and hosts during telia stage. The latter is demicyclic and autoecious on *Distylium*, cylindrical telia are similar to *N. sycopsis-sinensis* (Figure 4), and two-celled teliospores with thick walls are similar to *N. corylopsidis* and *N. hamamelidis* (Figure 5; Hiratsuka et al., 1992). However, teliospores of the latter are one-celled with elongated pedicel-like intercalary cells, while *P. sakamotoi* produces two-celled teliospores without intercalary cells (Hiratsuka, 1942). An autoecious and demicyclic life cycle can also distinguish it from similar species. No molecular data have been used to analyze the relationship between these two species and *Novopuccinia* on Hamamelidaceae, although we may assume that they are closely related to *N. corylopsidis* and *N. hamamelidis* based on the observation of similar teliospores, hosts of spermogonial/aecial stages, and distribution. Further investigations will confirm this conjecture.

## Key to Rust Fungal Species That Were Recorded on the Host Family Hamamelidaceae in Asia

1. Life cycle macrocyclic, heteroecious …………………………………… 2
1. Life cycle microcyclic or demicyclic and autoecious …………… 3
  2. Teliospores two-celled, pedicellate, wall apically prominently elongated; spermogonial and aecial stages on *Corylopsis* (Trib. Corylopsideae); distributed in Japan …………………………………………………….. *Puccinia sasicola*
  2. Teliospores two-celled, pedicellate, wall mostly unilaterally thickened; spermogonial and aecial stages on *Hamamelis* (Trib. Hamamelideae); distributed in Japan …………………………………….. *Novopuccinia hamamelidis*
3. Telia columnar; teliospore one-celled, catenulate; only telia produced on *Sycopsis* (Trib. Fothergilleae); distributed in China ……………………………………. *Novopuccinia sycopsis-sinensis*
3. Telia pulvinate …………………………………………………………………… 4
4. Teliospores two-celled, pedicellate, wall mostly unilaterally thickened; only telia produced on *Corylopsis* (Trib. Corylopsideae) and *Sycopsis* (Trib. Distylteae); distributed in China …………………………………………… *Novopuccinia corylopsidis*
4. Teliospores two-celled, pedicellate, wall evenly thickened at sides; aecia and telia produced on *Distylium* (Trib. Fothergilleae); distributed in Japan ………………………. *Puccinia sakamotoi*

## Data Availability Statement

The datasets presented in this study can be found in online repositories. The names of the repository/repositories and accession number(s) can be found below: https://www.ncbi.nlm.nih.gov/genbank/ MN943642, MN943643, MW136693, MW136697, MW386782, MW386783, MW394517.

## Author Contributions

YLa and CT conceived and designed the experiments. YLu, BC, and WL performed the experiments. YLu and YO analyzed the data. YLu wrote the manuscript. YO and YLa revised and approved the final version of the manuscript. All authors contributed extensively to the work presented in this manuscript.

## Conflict of Interest

The authors declare that the research was conducted in the absence of any commercial or financial relationships that could be construed as a potential conflict of interest. The reviewer PZ declared a shared affiliation with one of the authors BC to the handling editor at the time of review.

## Footnotes

1 http://www.indexfungorum.org

2 http://www.efloras.org/

3 http://mafft.cbrc.jp/alignment/server/

4 www.treebase.org
