# Supplementary figures and images for: Taxonomy and Phylogeny of Rust Fungi on Hamamelidaceae
Source: Front Microbiol. 2021 Apr 28;12:648890. doi: 10.3389/fmicb.2021.648890 (PMC8115210; doi:10.3389/fmicb.2021.648890)

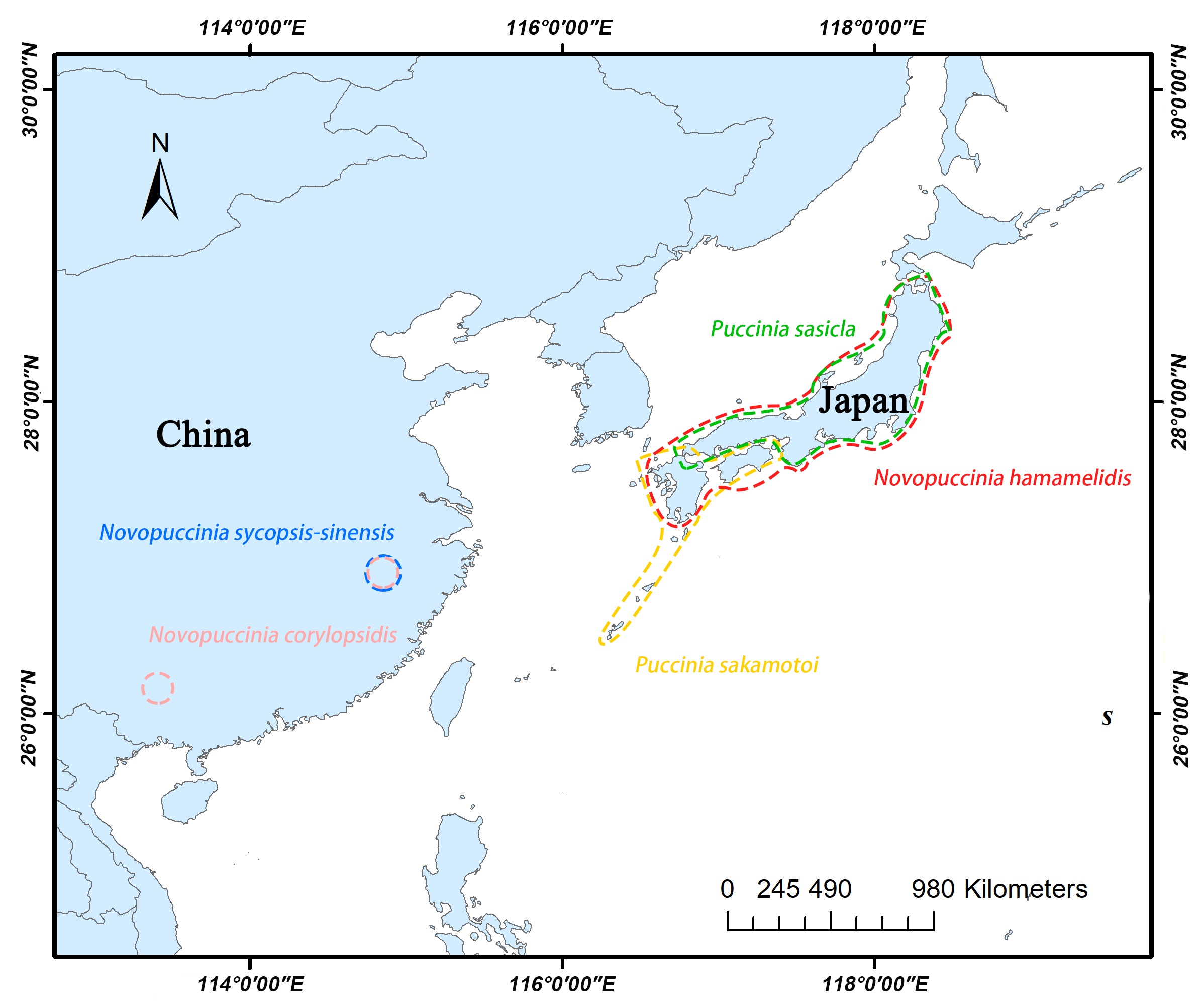

Supplement: Supplementary file 1 [file Image_1.tif]
