# Supplementary material for: Taxonomy and Phylogeny of Rust Fungi on Hamamelidaceae
Source: Front Microbiol. 2021 Apr 28;12:648890. doi: 10.3389/fmicb.2021.648890 (PMC8115210; doi:10.3389/fmicb.2021.648890)
Supplement: Supplementary file 2 [file Table_1.DOCX]

**Table S1.** Collections in Pucciniales used in phylogenetic analysis, locality, host, GenBank number and origin. Specimens from this study are in bold

| **Taxa** | **Accession number** | **Host** | **GenBank accession no.** | | **References** |
| --- | --- | --- | --- | --- | --- |
|  |  |  | **LSU rDNA** | **ITS** |  |
| *Achrotelium ichnocarpi* | BRIP:55634 | *Ichnocarpus frutescens* | KT199393 | – | McTaggart et al. 2016 |
| *Allodus podophylli* | BPI 842277 | *Podophyllum peltatum* | DQ354543 | – | Aime 2006 |
| *Aplopsora nyssae* | BPI 877823 | *Nyssa sylvatica* | MW049244 | – | Aime and McTaggart 2020 |
| *Araucaromyces fragiformis* | BRIP 68996 | *Agathis robusta* | MW049245 | – | Aime and McTaggart 2020 |
| *Austropuccinia psidii* | BRIP:57793 | *Rhodamnia angustifolia* | KF318449 | – | GS Pegg et al. 2014 |
| *Blastospora smilacis* | PUR N270 | *Smilax sieboldii* | DQ354568 | – | Aime 2006 |
| *Catenulopsora flacourtiae* | PUR N13865 | *Flacourtia indica* | MW049248 | – | Aime and McTaggart 2020 |
| *Cephalotelium macowaniana* | PREM61222 | *Vachellia karroo* | MG946007 | – | Ebinghaus and Begerow 2018 |
| *Ceratocoma jacksoniae* | BRIP:57762 | *Daviesia* sp. | KT199394 | – | McTaggart et al. 2016 |
| *Chardoniella gynoxidis* | – | *–* | GU936636 | – | Unpublished |
| *Chardoniella gynoxidis* | R31 | *Ageratina popayanensis* | EU851132 | – | Unpublished |
| *Chardoniella gynoxidis* | R15 | *Gynoxys* sp. | MW049250 | – | Aime and McTaggart 2020 |
| *Chrysocelis lupini* | PUR N11562 | *Lupinus* sp. | MW049251 | – | Aime and McTaggart 2020 |
| *Chrysocyclus sp.* | ZP-R905 | *–* | MK518700 | MK518996 | Unpublished |
| *Coleopuccinia sinensis* | BJFC-R02506 | *–* | MF802285 | – | Cao et al. 2018 |
| *Coleosporium senecionis* | PDD 98309 | *Senecio* sp. | KJ716348 | – | Padamsee and McKenzie 2014 |
| *Cronartium flaccidum* | PUR N16561 | *Vincetoxicum hirundinaria* | MW049253 | – | Aime and McTaggart 2020 |
| *Crossopsora ziziphi* | BPI 877877 | *Ziziphus mucronata* | MG744558 | – | Souza et al. 2018 |
| *Cumminsiella mirabilissima* | WM 1351 | *Mahonia aquifolium* | AF426206 | – | Maier 2003 |
| *Cumminsiella mirabilissima* | BPI 871101 | *Mahonia aquifolium* | DQ354531 | – | Aime 2006 |
| *Dasyspora gregaria* | ZT Myc 3397 | *Xylopia cayennensis* | JF263477 | – | Beenken et al. 2012 |
| *Dasyspora guianensis* | ZT Myc 3413 | *Xylopia benthamii* | JF263479 | JF263479 | Beenken et al. 2012 |
| *Desmella aneimiae* | BRIP 60995 | *Nephrolepis hirsutula* | KM249867 | – | McTaggart et al. 2014 |
| *Didymopsora solani-argentei* | PUR N3728 | *Solanum argentinum* | MW049254 | – | Aime and McTaggart 2020 |
| *Dietelia duguetiae* | PUR 87978 | *Duguetia furfuracea* | KM217365 | KM217365 | Unpublished |
| *Edythea quitensis* | QCAM6453 | *Berberis hallii* | MG596499 | – | Unpublished |
| *Elateraecium salaciicola* | PUR F17677 | *Salacia* sp. | MW049257 | – | Aime and McTaggart 2020 |
| *Endophylloides portoricensis* | BPI 844288 | *Mikania micrantha* | DQ354516 | – | Aime 2015 |
| *Endophyllum euphorbiae-sylvaticae* | HeRB C-82 | *Euphorbia amygdaloides* | AF426200 | – | Maier 2003 |
| *Endophyllum sempervivi* | TUB 14957 | *Sempervivum tectorum* | DQ917747 | – | Maier 2007 |
| *Endoraecium acaciae* | BPI 871098 | *Acacia koa* | DQ323916 | – | Scholle and Aime 2006 |
| *Eocronartium muscicola* | MIN796447 | – | AF014825 | – | Direct Submission |
| *Gerwasia rubi* | BRIP:58369 | *Rubus* sp. | KT199397 | – | McTaggart et al. 2016 |
| *Gymnosporangium clavariiforme* | BRIP 59471 | *Crataegus* sp. | MW049261 | – | Aime and McTaggart 2020 |
| *Gymnosporangium sabinae* | TNM F0030477 | *Pyrus communis* | KY964764 | – | Shen et al. 2018 |
| *Gymnoconia interstitialis* | BPI:747600 | *Rubus allegheniensis* | JF907677 | – | Yun et al. 2011 |
| *Gymnotelium blasdaleanum* | U1469 | *Amelanchier alnifolia* | MG907218 | – | Aime et al. 2018 |
| *Hamaspora longissima* | BPI 871506 | *Rubus ludwigii* | MW049262 | – | Aime and McTaggart 2020 |
| *Hapalophragmium derridis* | PUR N16494 | Fabaceae | MW049263 | – | Aime and McTaggart 2020 |
| *Kuehneola uredinis* | BPI 871104 | *Rubus argutus* | DQ354551 | – | Aime 2006 |
| *Lipocystis caesalpiniae* | BPI 863966 | *Mimosa ceratonia* | MW049265 | – | Aime and McTaggart 2020 |
| *Macruropyxis fraxini* | ZT Myc 56551 | *Fraxinus platypoda* | KP858145 | – | Beenken and Wood 2015 |
| *Masseeëlla capparis* | BRIP:56844 | *Flueggea virosa* | JX136798 | – | Unpublished |
| *Melampsoridium betulinum* | BPI 871107 | *Alnus* sp. | DQ354561 | – | Aime 2006 |
| *Melampsora euphorbiae* | BPI 863501 | *Euphorbia macroclada* | DQ437504 | – | Unpublished |
| *Melampsorella caryophyllacearum* | PUR 82 | *Caryophyllaceae Cerastium* | MG907233 | – | Aime et al. 2018 |
| *Mikronegeria fagi* | PUR N16373 | *Lophozonia obliqua* | MW049267 | – | Aime and McTaggart 2020 |
| *Milesia polypodii* | KR-M-0043190 | *Polypodium vulgare* | MK302190 | – | Bubner et al. 2019 |
| *Milesina kriegeriana* | KR-M-0048480 | *Dryopteris dilatata* | MK302207 | – | Bubner et al. 2019 |
| *Miyagia pseudosphaeria* | PDD:97677 | *Sonchus oleraceus* | KX985753 | KX985753 | Padamsee and McKenzie 2017 |
| *Naohidemyces vaccinii* | BPI 871754 | *Vaccinium ovatum* | DQ354563 | – | Aime 2006 |
| *Neoolivea tectonae* | PUR N15331 | *Tectona grandis* | MW049282 | – | Aime and McTaggart 2020 |
| *Neophysopella ampelopsidis* | IBA-8597 | *Ampelopsis brevipedunculata* | AB354738 | – | Chatasiri and Ono 2008 |
| *Newinia heterophragmatis* | PUR N16505 | *Kigelia cf. africana* | MW049271 | – | Aime and McTaggart 2020 |
| *Nothoravenelia japonica* | HMJAU8598 | – | MK296509 | – | Direct Submission |
| *Novopuccinia corylopsidis* | BJFC-R02977 | *Sycopsis sinensis* | MH184512 | MH178664 | Tao et al. 2020 |
| ***Novopuccinia corylopsidis*** | **BJFC-R02978** | ***Sycopsis sinensis*** | **MW136697** | **MW136693** | **This study** |
| ***Novopuccinia hamamelidis*** | **BJFC-R3798（IBAR6666）** | ***Hamamelis japonica*** | **MW394517** | – | **This study** |
| ***Novopuccinia hamamelidis*** | **BJFC-R3799（IBAR8075）** | ***Hamamelis japonica*** | **MW394518** | – | **This study** |
| ***Novopuccinia sycopsis-sinensis*** | **BJFC-R02976** | ***Sycopsis sinensis*** | **MN943642** | **MW386782** | **This study** |
| ***Novopuccinia sycopsis-sinensis*** | **BJFC-R02983** | ***Sycopsis sinensis*** | **MN943643** | **MW386783** | **This study** |
| *Nyssopsora echinata* | KR0012164 | *Meum athamanticum* | MW049272 | – | Aime and McTaggart 2020 |
| *Ochropsora ariae* | KR0015027 | *Anemone nemorosa* | MW049273 | – | Aime and McTaggart 2020 |
| *Phakopsora pachyrhizi* | BRIP:56941 | *Desmodium* sp. | KP729475 | – | Maiera et al. 2015 |
| *Phragmidium mucronatum* | BRIP 60097 | *Rosa rubiginosa* | MW049275 | – | Aime and McTaggart 2020 |
| *Pileolaria brevipes* | MCA3477 | *Anacardiaceae toxicodendron* | MG907216 | – | Aime et al. 2018 |
| *Pileolaria shiraiana* | BRIP 58344 | *Rhus japonica* | KJ651957 | – | Doungsa-ard et al. 2015 |
| *Pileolaria terebenthi* | – | *Pistacia* sp. | KY796222 | – | Ishaq et al. 2020 |
| *Prospodium appendiculatum* | BPI 879956 | *Tecoma stans* | MW049276 | – | Aime and McTaggart 2020 |
| *Puccinia allii* | BRIP:59724 | *Allium sativum* | KM249851 | KM249851 | McTaggart et al. 2016 |
| *Puccinia andropogonis* | HSZ0264 | *Lupinus perennis* | DQ344519 | DQ344519 | Szabo 2006 |
| *Puccinia andropogonis var. onobrychidis* | – | *Baptisia australis* | EF583818 | EF583818 | Unpublished |
| *Puccinia bartholomaei* | – | *Asclepias viridis* | EF583820 | EF583820 | Unpublished |
| *Puccinia chrysanthemi* | PcCA1 | *–* | EU816926 | EU816926 | Pedley 2009 |
| *Puccinia coronata* | 73MN873 | *Elytrigia repens* | DQ414723 | DQ414723 | Szabo 2006 |
| *Puccinia emaculata* | BPI 851570 | *Panicum capillare* | KX190848 | KX190848 | Demer et al. 2017 |
| *Puccinia graminis* | HSZ0804 | *Lolium perenne* | DQ355455 | DQ355455 | Szabo 2006 |
| *Puccinia graminis* | BRIP:60137 | *Glyceria maxima* | KM249852 | – | McTaggart et al. 2016 |
| *Puccinia hemerocallidis* | BRIP:53476 | *Hemerocallis* sp. | KM249855 | KM249855 | McTaggart et al. 2016 |
| *Puccinia jaceae* | OL 33481 | *Centaurea macrocephala* | KX468974 | KX468974 | Petrželová et al. 2017 |
| *Puccinia lagenophorae* | HSZ0715 | *Senecio* sp. | KM096425 | KM096425 | Unpublished |
| *Puccinia mixta* | BRIP 61576 | *Allium schoenoprasum* | KU296893 | KU296893 | McTaggart et al. 2016 |
| *Puccinia mysuruensis* | HSZ2119 | *Psychotria nervosa* | KC847089 | KC847089 | Mahadevakumar et al. 2016 |
| *Puccinia novopanici* | BPI 747673 | *Panicum virgatum* | KX190874 | KX190874 | Demer et al. 2017 |
| *Pucciniosira pallidula* | BPI 863541 | *Triumfetta semitriloba* | DQ354534 | – | Aime 2006 |
| *Puccinia polysora* | HSZ1879 | *Zea mays* | HQ189433 | HQ189433 | Crouch and Szabo 2011 |
| *Puccinia porri* | BRIP 64600 | *Allium porrum* | KY849820 | KY849820 | McTaggart et al. 2017 |
| *Puccinia setariae* | BPI 188745 | *Setaria imberbis* | KX190907 | KX190907 | Demer et al. 2017 |
| *Puccinia veronicae-longifoliae* | BPI 841971 | *Veronica spicata* | JQ627617 | JQ627617 | Stoxen et al. 2013 |
| *Puccinia wiehei* | BPI 111530 | *Setaria sphacelata* var*. splendida* | KX190913 | KX190913 | Demers et al. 2017 |
| *Pucciniastrum epilobii* | PUR N11088 | *Chamaenerion angustifolium* | MW049277 | – | Aime and McTaggart 2020 |
| *Puccorchidium polyalthiae* | ZT HeRB 251 | *Polyalthia longifolia* | JF263493 | – | Beenken et al. 2012 |
| *Rogerpetersonia torreyae* | U808 | *Torreya californica* | MG907207 | – | Aime et al. 2018 |
| *Rossmanomyces pyrolae* | 390CHP-PCG-VF1 | – | FJ666456 | – | Vialle et al. 2009 |
| *Skierka diploglottidis* | BRIP 59646 | *Dictyoneura obtusa* | MW049278 | – | Aime and McTaggart 2020 |
| *Skierka robusta* | BPI 879954 | *Rhoicissus rhomboidea* | MW049279 | – | Aime and McTaggart 2020 |
| *Sphaerophragmium acaciae* | BRIP 56910 | *Albizia* sp. | KJ862350 | – | McTaggart et al. 2015 |
| *Sphenorchidium xylopiae* | NY s.n. | *Xylopia aethiopica* | KM217355 | – | Beenken and Wood 2015 |
| *Stereostratum corticioides* | ZP-R1001 | *Choerospondias* sp. | MK518727 | MK519023 | Unpublished |
| *Stereostratum corticioides* | ZP-R1003 | *Choerospondias* sp. | MK518707 | MK519002 | Unpublished |
| *Stereostratum corticioides* | BPI 842314 | *Bambusa* sp. | MW049281 | – | Aime and McTaggart 2020 |
| *Thekopsora areolata* | *–* | *Picea engelmannii* | KJ546894 | – | Unpublished |
| *Trachyspora intrusa* | BPI 843828 | *Alchemilla vulgaris* | DQ354550 | – | Aime 2006 |
| *Tranzschelia mexicana* | KR-M-0040855 | *Prunus salicifolia* | KP308391 | – | Blomquist et al. 2015 |
| *Uromyces aemulus* | HSZ0345 | *–* | AF511081 | AF511081 | Anikster et al. 2004 |
| *Uromyces appendiculatus* | BRIP 60020 | *Phaseolus vulgaris* | KM249870 | – | McTaggart et al. 2014 |
| *Uromyces appendiculatus* var. *crassitunicatus* | BRIP 60929 | *Macroptilium atropurpureum* | KU296911 | KU296911 | McTaggart et al. 2016 |
| *Uromyces betae* | BPI 910289 | *Beta* sp. | KY764187 | KY764187 | Unpublished |
| *Uromyces ciceris-arietini* | BPI 879192 | *Cicer arietinum* | GU058030 | GU058030 | Dixon et al. 2010 |
| *Uromyces galegae* | BPI 863535 | *Galega officinalis* | DQ250133 | DQ250133 | Unpublished |
| *Uromyces limonii* | BPI 910295 | *Limonium sinuatum* | KY764194 | KY764194 | Unpublished |
| *Uromyces reichertii* | – | *–* | AF511084 | AF511084 | Anikster et al. 2004 |
| *Uromyces scillarum* | YA3464 | *–* | AF511085 | AF511085 | Anikster et al. 2004 |
| *Uromyces transversalis* | BPI 871558 | *Gladiolus* sp. | KY764204 | KY764204 | Unpublished |
| *Xenodochus carbonarius* | PUR N15566 | *Sanguisorba officinalis* | MW049289 | – | Aime and McTaggart 2020 |
